# Integrative Bioinformatic Analyses of Global Transcriptome Data Decipher Novel Molecular Insights into Cardiac Anti-Fibrotic Therapies

**DOI:** 10.3390/ijms21134727

**Published:** 2020-07-02

**Authors:** Maximilian Fuchs, Fabian Philipp Kreutzer, Lorenz A. Kapsner, Saskia Mitzka, Annette Just, Filippo Perbellini, Cesare M. Terracciano, Ke Xiao, Robert Geffers, Christian Bogdan, Hans-Ulrich Prokosch, Jan Fiedler, Thomas Thum, Meik Kunz

**Affiliations:** 1 Chair of Medical Informatics, Friedrich-Alexander University (FAU) of Erlangen-Nürnberg, 91058 Erlangen, Germany; maximilian.fuchs@fau.de (M.F.); hans-ulrich.prokosch@fau.de (H.-U.P.); 2 Functional Genomics and Systems Biology Group, Department of Bioinformatics, University of Würzburg, 97074 Würzburg, Germany; 3 Institute of Molecular and Translational Therapeutic Strategies (IMTTS), Hannover Medical School, 30625 Hannover, Germany; Kreutzer.Fabian@mh-hannover.de (F.P.K.); Mitzka.Saskia@mh-hannover.de (S.M.); Just.Annette@mh-hannover.de (A.J.); Perbellini.Filippo@mh-hannover.de (F.P.); Xiao.Ke@mh-hannover.de (K.X.); Fiedler.Jan@mh-hannover.de (J.F.); 4 Medical Center for Information and Communication Technology, Universitätsklinikum Erlangen, 91054 Erlangen, Germany; lorenz.kapsner@uk-erlangen.de; 5 National Heart and Lung Institute, Imperial College London, Du Cane Road, London W12 0NN, UK; c.terracciano@imperial.ac.uk; 6 Helmholtz Centre for Infection Research, 38124 Braunschweig, Germany; robert.geffers@helmholtz-hzi.de; 7 Mikrobiologisches Institut—Klinische Mikrobiologie, Immunologie und Hygiene, Universitätsklinikum Erlangen and Friedrich-Alexander Universität (FAU) Erlangen-Nürnberg, 91054 Erlangen, Germany; Christian.Bogdan@uk-erlangen.de; 8 REBIRTH Center for Translational Regenerative Medicine, Hannover Medical School, 30625 Hannover, Germany

**Keywords:** big data, transcriptomics, integrative bioinformatics, algorithm, web application, natural compounds, miRNAs, cardiac fibrosis

## Abstract

Integrative bioinformatics is an emerging field in the big data era, offering a steadily increasing number of algorithms and analysis tools. However, for researchers in experimental life sciences it is often difficult to follow and properly apply the bioinformatical methods in order to unravel the complexity and systemic effects of omics data. Here, we present an integrative bioinformatics pipeline to decipher crucial biological insights from global transcriptome profiling data to validate innovative therapeutics. It is available as a web application for an interactive and simplified analysis without the need for programming skills or deep bioinformatics background. The approach was applied to an ex vivo cardiac model treated with natural anti-fibrotic compounds and we obtained new mechanistic insights into their anti-fibrotic action and molecular interplay with miRNAs in cardiac fibrosis. Several gene pathways associated with proliferation, extracellular matrix processes and wound healing were altered, and we could identify micro (mi) RNA-21-5p and miRNA-223-3p as key molecular components related to the anti-fibrotic treatment. Importantly, our pipeline is not restricted to a specific cell type or disease and can be broadly applied to better understand the unprecedented level of complexity in big data research.

## 1. Introduction

Bioinformatics is an emerging field in the big data era, offering a steadily increasing number of algorithms and analysis tools. To obtain full insights into the systemic and pathological effects of omics data, multiple global datasets have to be integrated and analyzed with different methods on distinct levels of regulation.

Since researchers often use RNA-Seq as an introduction to the analysis of omics data, various web services such as g:Profiler [1] and DAVID ([2] (see [3] for an overview) are available that allow the analysis of differentially expressed genes derived from upstream raw read count data analysis. These individual downstream analyses can be applied depending on the research questions and experimental settings (e.g., functional enrichment analysis, molecular network analysis).

Commonly used methods for gene-level differential expression analysis of raw read count data are DESeq2, edgeR, and limma [4], and are available as Galaxy pipeline and R package. On the other hand, database tools such as Expression Atlas [5], RNASeq-er API [6], and The Cancer Genome Atlas (TCGA) offer analyses including annotation, normalization, and differential expression analysis of public RNA-Seq data. However, such bioinformatics pipelines tend to be quite complicated as advanced statistics and programming tasks need to be combined, which remains challenging for researchers in experimental life sciences without background in bioinformatics. Moreover, they are restricted to a specific disease and/or did not allow analysis of their own experimental data. These characteristics limit them for an easy-to-use broad application.

Cardiovascular diseases (CVD) represent the leading cause of death worldwide with over 17 million deaths estimated for 2015 [7], underlining the need for systematic research on novel therapeutic approaches. One of the main characteristics of CVD is heart failure, a life threatening condition that affects heart function and often results in increased stiffness of the heart [8,9]. This stiffening of cardiac tissue is mainly caused by activation and proliferation of cardiac fibroblasts, which lead to accumulation of excess extracellular matrix (ECM) in a process termed cardiac fibrosis. Unfortunately, therapeutic options for cardiac fibrosis patients are still limited or absent [8].

A potential treatment strategy counteracting the increasing stiffness of the heart is to inhibit the proliferation of human cardiac fibroblasts (HCF), the main effector cells of cardiac fibrosis. In this context, we previously showed that therapeutic antagonism of miRNA-21 inhibited cardiac fibrosis in various cardiac disease models via modulating fibroblast ERK–MAP kinase activity [10,11]. Furthermore, we observed anti-fibrotic activity in kidney diseases [12]. In addition to single microRNA (miRNA) targeting, inhibition of the fibroblast-enriched long non-coding RNA Meg3 was able to prevent cardiac fibrosis through repression of matrix metalloproteinase-2 [13]. Besides the use of non-coding RNA inhibitors, we recently demonstrated that the selected natural compounds bufalin and lycorine were effective in counteracting detrimental cardiac fibrosis in various animal models [14]. Thus, understanding the molecular action of anti-fibrotic drugs and its functional interplay with miRNAs will guide the future treatment of cardiac fibrosis.

Here, we developed a novel integrated bioinformatics pipeline for an efficient global transcriptome analysis. A living multicellular ex vivo cardiac model preparation was treated with cytostatic small molecule similars of lycorine (lyco-s) and bufalin (buf-s) and the bioinformatic pipeline was applied to the generated RNA-Seq and miRNA-Seq dataset. The aim was to identify the mechanism of their anti-fibrotic activity and their interplay with miRNAs.

## 2. Results

### 2.1. Bioinformatics RNA-Seq Analysis Showed Deregulation of Lyc-s but Not of Buf-s Compared to DMSO

Living myocardial slices were prepared from Sprague Dawley rat hearts and cultured in vitro by applying biomimetic electromechanical stimulation [15,16]. The myocardial slices were treated with lyco-s or buf-s for 24 h (*n* = 3). Total RNA was isolated and used for next-generation RNA sequencing.

**Figure 1:**
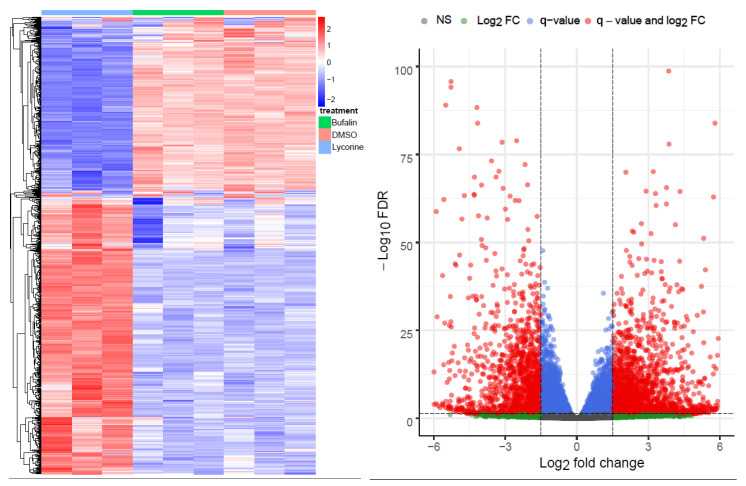
Overview of the RNA-Seq. (**Left**) The heatmap shows the top 1000 highly variant genes (x-axis: samples of lyco-s, buf-s and DMSO, *n* = 3; in red: upregulation, in blue: downregulation). (**Right**) The volcano plot shows the differential expression analysis of the comparison lyco-s vs. DMSO (genes above thresholds highlighted by coloring).

The principal component analysis (PCA) and multidimensional scaling (MDS) plot showed similar profiles of buf-s and dimethyl sulfoxide (DMSO), whereas lyco-s grouped alone (Figure S1 Top and Middle). The top 1000 highly variant genes are shown in Figure 1 (left panel). Differential gene analysis (logFC > 1.5, <−1.5; adj. *p* < 0.05; differentially expressed genes (DEGs) in Table S1) results in 1836 upregulated and 1343 downregulated genes between lyco-s and DMSO (Figure 1, right panel), and 2225 upregulated and 1501 downregulated genes between lyco-s and buf-s (Figure S1 Bottom). Notably, the comparison between buf-s and DMSO did not yield significant differentially expressed genes. Mapping of the DEGs showed an overlap of 2659 genes, whereas 520 DEGs showed unique expression in lyco-s vs. DMSO and 1067 DEGs in lyco-s vs. buf-s treated samples (detailed mapping in Table S1).

### 2.2. Functional Analysis Revealed Enrichment of Processes Involved in Cardiac Fibrosis

**Figure 2:**
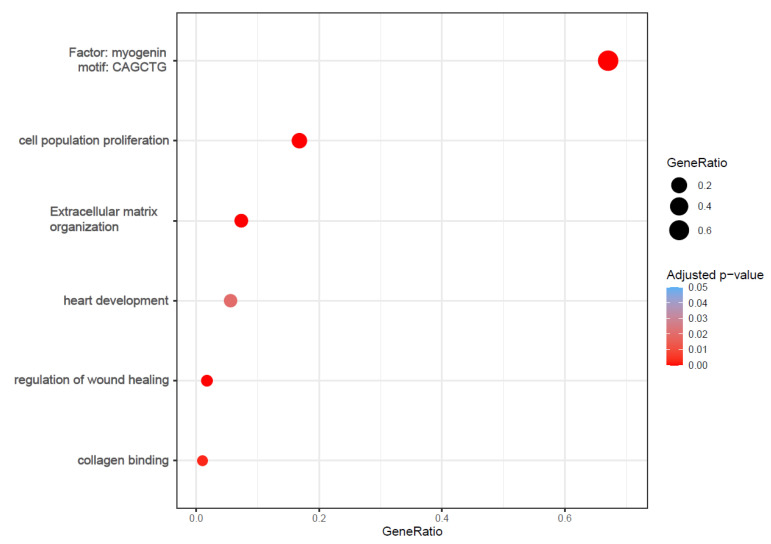
Bubble plots of functional enrichment analysis. The plot shows selected processes enriched in the up- and downregulated genes after treatment with lyco-s. Bubble size represents the ratio between the genes connected to a process and the overall query size. The color scale shows the adjusted *p*-value (Table S2 for whole functional enrichment analysis).

We focused on the gene regulation induced by lyco-s vs. DMSO treatment. The functional analysis of the significantly up- and downregulated genes emphasized enrichment of several processes involved in cardiac fibrosis, such as cell proliferation, wound healing, ECM organization as well as collagen binding (Figure 2; whole functional enrichment analysis in Table S2).

### 2.3. Molecular Network Analysis Identified miRNA-21-5p and miRNA-223-3p as Key Interaction Partners around the DEGs

**Figure 3:**
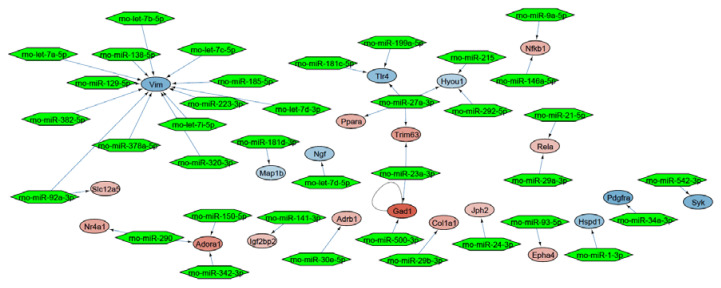
miRNA interaction network around the DEGs. Every network node represents a DEG, while the edges represent interactions. The color scaling shows the deregulation of genes (blue: downregulated, red: upregulated). Green hexagons represent potential miRNA interaction partners.

Mapping of the DEGs to the whole rat interactome resulted in a molecular interaction network of 423 nodes and 113 edges (Figure S2). In this network, we identified a functional regulated miRNA interaction subnetwork of 37 miRNAs around 21 DEGs (Figure 3).

**Figure 4:**
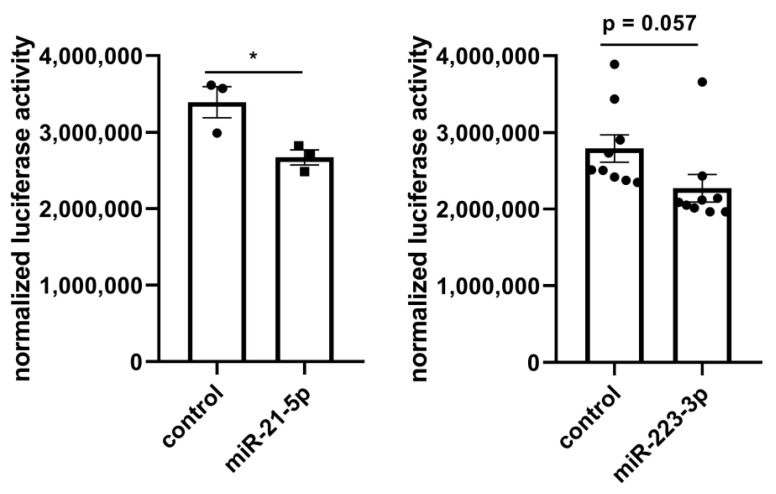
In vitro luciferase reporter system to validate predicted miRNA interaction. (**Left**) Luciferase reporter system to assess the binding of miRNA-21-5p to *RELA* 3′-UTR. Co-transfection of a *RELA* luciferase expression system with miRNA (control, miRNA-21-5p) highlights the regulatory role of miRNA-21-5p. Enhanced miRNA-21-5p expression efficiently repressed luciferase reporter gene expression compared to control levels. *n* = 3, * *p* < 0.05, unpaired t-test. (**Right**) Luciferase reporter system to assess the binding of miRNA-223-3p to *VIM* 3’-UTR. Co-transfection of a *VIM* luciferase expression system with miRNA (control, miRNA-223-3p) indicates the regulatory role of miRNA-223-3p. Synthetic miRNA-223-3p overexpression efficiently repressed luciferase reporter gene expression compared to control levels. *n* = 9, unpaired *t*-test.

The identified 37 miRNAs were validated with a miRNA-Seq dataset after treatment with lyco-s obtained using the same myocardial slices (Table S3). Out of the 9 significantly deregulated miRNAs in the validation miRNA-Seq (adj. *p* < 0.05), downregulation of miRNA-21-5p (logFC = −1.22, adj. *p* = 9.71 × 10^−8^) and miRNA-223-3p (logFC = −1.90, adj. *p* = 9.71 × 10^−8^) after treatment with lyco-s could be confirmed. The potential regulation of the direct miRNA-21-5p target gene *RELA (RELA proto-oncogene, NF-κB subunit)* was additionally validated in a luciferase reporter system (Figure 4, left panel). Subcloning of *RELA* 3’UTR (untranslated region) and respective miRNA-21 binding sites to a luciferase-based expression system in vitro revealed that sustained miRNA-21 expression can repress *RELA*. Another validation was performed for miRNA-223-3p binding to *vimentin* (*VIM)* 3′-UTR as suggested by in silico analysis, indicating a similar miRNA-based regulation of mRNA expression (Figure 4, right panel). Thus, the herein presented miRNA interaction network approach has a great prediction strength on miRNA-dependent regulation in our experimental setting of ex vivo cardiac tissue.

## 3. Discussion

Integrative bioinformatics analyses of global datasets and different interaction levels offer insights into the systemic and pathological effects of diseases such as cardiac fibrosis. We developed a novel bioinformatics pipeline for the integrated global transcriptome analysis, enabling to bridge the gap between raw read count and functional molecular interaction analysis. The pipeline is available as a Shiny web application for an interactive and simplified analysis without the need for programming skills or deep bioinformatics background. Here, the pipeline has been used for the analysis of a rat dataset from an ex vivo cardiac fibrosis model. However, it is not restricted to a specific cell-type or disease and can be broadly applied in big data research.

We recently proved that reduction of proliferation in cardiac fibroblasts using natural compounds is a novel therapy to prevent cardiac fibrosis [14]. Here, we observed an anti-fibrotic effect for a small molecular similar of the natural compound lycorine compared to DMSO in our ex vivo cardiac fibrosis model, whereas a similar of bufalin turned out to be ineffective. We hypothesize that the molecules directly targeted by the two small molecule similars differ from each other and thereby exert distinct effects leading to anti-fibrotic gene reprogramming.

The functional enrichment analysis showed repression of proliferative pathways as key molecular effects of the lyco-s treatment. Under pathological conditions in the heart, quiescent tissue-resident cardiac fibroblasts are activated, start proliferating, and migrate into the injured tissue. During cardiac remodeling, these fibroblasts differentiate into alpha-smooth muscle actin (αSMA)-expressing myofibroblasts (logFC = 1.3 after lyco-s) to stabilize the injured tissue [17]. Indeed, both natural compounds, lycorine and bufalin, were previously selected due to their anti-proliferative and migration-inhibitory effects on cardiac fibroblasts [14].

Additionally, our analysis pipeline predicted changes associated with ECM processes, collagen binding, heart development, and wound healing. The common effector molecule for cardiac fibroblast proliferation and differentiation into myofibroblasts is the cytokine transforming growth factor (TGF)-β [18]. After initial stabilization, myofibroblasts trigger remodeling and secrete additional ECM to build a long-term scar [19,20]. Furthermore, there is a functional enrichment for binding motifs of myogenin, indicating a potential anti-fibrotic regulation by transcription factors associated with muscle cell development and wound healing [21].

The molecular interaction analysis provided mechanistic insights into the anti-fibrotic action and revealed miRNA-21-5p and miRNA-223-3p as key downregulated miRNAs which we could validate with a miRNA-Seq dataset. We previously showed that cardiac fibrosis is regulated by miRNAs such as the pro-proliferative miRNA-21 that inhibits the direct target gene *sprouty RTK signaling antagonist 1* (*SPRY1*) which is an intrinsic inhibitor of growth factor signaling [10]. Selective knockdown of miRNA-21 by synthetic oligonucleotides has been established as an anti-fibrotic concept also for other organs besides the heart, including lung [22] and kidney [23]. Natural compound similar lyco-s potently suppressed miRNA-21 thereby underlining its anti-fibrotic mode of action. In our study, the interaction map identified *RELA* as a direct target gene of miRNA-21 which has been discussed as a therapeutic target in pulmonary fibrosis [24]. Whether *RELA* is of relevance in cardiac fibroblast biology potentially also via the modulation of growth factor signaling, as observed for *SPRY1*, needs to be determined in future studies. Of interest, miRNA-21 dependent regulation of *RELA* was confirmed in our experimental setup, thus underlining the link of in silico prediction to genuine miRNA biology and mode of action.

In addition to miRNA-21 and its pro-fibrotic function we identified miRNA-223 as another deregulated miRNA candidate in our experimental setting. In the context of myocardial tissue and fibroblast function a role of miRNA-223 has been unknown to date. However, there is published evidence that synthetic miRNA-223 oligonucleotides ameliorated liver fibrosis [25]. Thus, we speculate that miRNA-223-dependent repression of *Vim* also contributes to fibroblast survival in our ex vivo model of myocardial slices.

However, our findings need to be confirmed on a single cell level as the analyzed rat myocardial tissue consists of different cell types. Single cell RNA sequencing approaches may be of interest for upcoming studies in whole heart tissue of rats as recently performed for the transcriptome of 20 different mouse organs [26].

Taken together, the downregulation of miRNA-21 and miRNA-223 appears to be linked to fibroblast signaling. Our findings underline the importance of in silico omics data analysis in pre- clinical experimental model systems to validate innovative therapeutics.

## 4. Materials and Methods

### 4.1. Animal Experiments

Animal experiments to prepare living myocardial slices were conducted at Imperial College London and were performed as licensed by the UK Home Office in accordance with the United Kingdom Animals (Scientific Procedures) Act 1986. The use of animals complied with institutional and national regulations and followed the guidelines established by the European Directive on the protection of animals used for scientific purposes (2010/63/EU).

The preparation and ex vivo culture of myocardial slices was performed as previously described [15,16]. Briefly, hearts of healthy rats were explanted, 300 µm thick living myocardial slices were generated using a high precision vibratome and cultured for 24 h in Medium 199 (SIGMA) + 3% Pen/Strep (Life Technologies, Carlsbad, CA, USA) in the presence of 45 nM buf-s, 340 nM lyco-s (Greenpharma S.A.S., Orléans, France) or 0.1% DMSO (SIGMA, Kanagawa, Japan) as solvent control in custom-made culture chambers under constant 1 Hz field stimulation (*n* = 3 for each condition). After culture, myocardial slices were snapfrozen in liquid nitrogen, homogenized in TriFast with a Precellys 24 (bertin instruments) and total RNA was isolated with miRNeasy kit (Qiagen, Hilden, Germany) as described [14].

The NEBNext Ultra II Directional RNA and Illumina TruSeq^®^ Small Library Prep Kit were applied to prepare the samples for subsequent deep sequencing on a NovaSeq 6000 (mRNA, Illumina, San Diego, CA, USA) or HiSeq 2500 (miRNA, Illumina) at HZI Braunschweig. Quantification of short reads was performed by HZI Braunschweig, whereas raw reads were mapped to the rat genome rno6.0 using RNA STAR [27] and annotated with transcriptome reference (Rnor_6.0 Ensembl) and miRBase v22 [28], respectively. Before mapping, the trim tool “Trim Galore!” was applied to remove the low quality reads with phred quality score less than 25. During the mapping with STAR, we set the MAPQ = 3 to keep reads that can be mapped maximally to two different loci in genome. The raw read counts were then derived applying featureCounts [29].

### 4.2. Bioinformatics Transcriptome Analysis Approach

**Figure 5:**
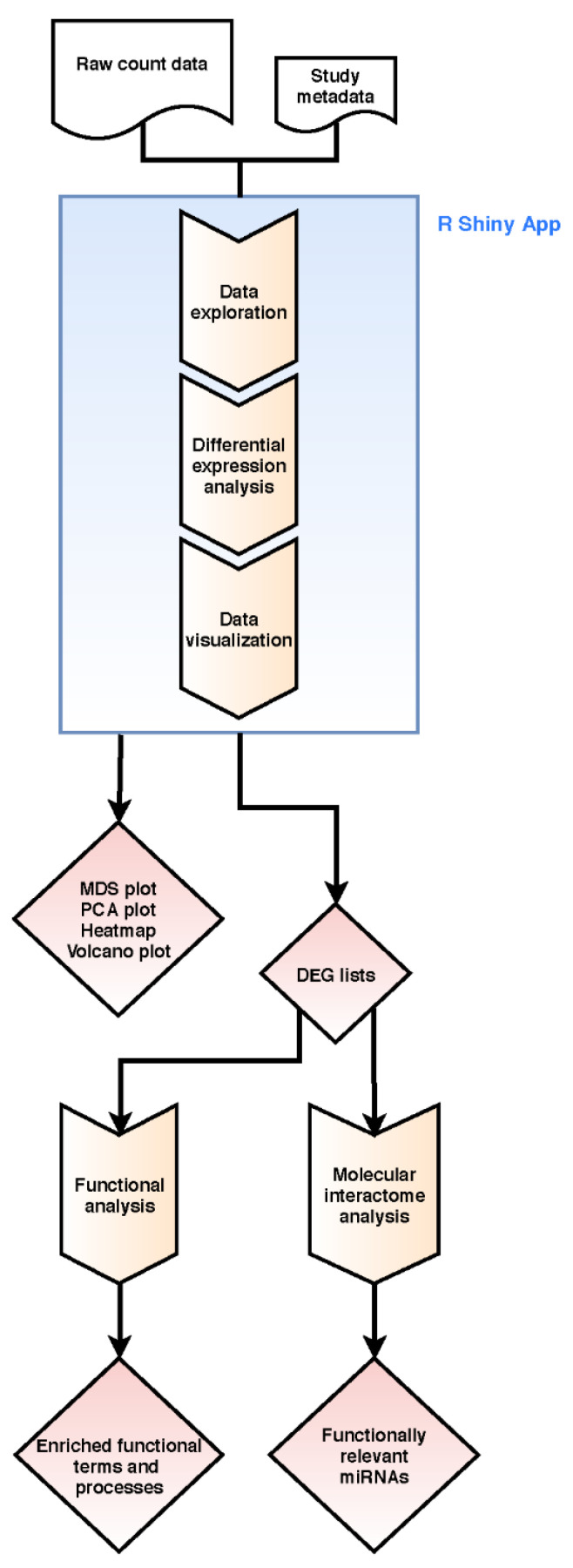
Workflow of our integrative bioinformatics analysis combining raw read count and functional molecular interactome analysis. Step boxes illustrate analysis steps, diamonds results/outcomes.

The workflow of our pipeline is shown in Figure 5 and applied to mRNA-Seq and miRNA-Seq count datasets. It combines read count data analysis with individual functional downstream analysis.

Raw read count data analysis includes state-of-the-art bioinformatics methods for filtering, normalization, batch effect detection, data visualization, and differential expression analysis using R version 3.6.3. Low-expressed genes with less than 10 summarized counts over all samples are filtered out. DESeq2 package version 1.26.0 [30] is used for logarithmic transformation of the data for data exploration. PCA and MDS data are generated from the transformed data with DESeq2 [30] and plotted using ggplot2 package version 3.3.0 [31].

Differential expression analysis is done using the DESeq2 [30] standard approach. Adjusted *p*-values are calculated using the Benjamini–Hochberg method [32] within DESeq2 [30]. Gene annotations are added to the result files using the Annotation.db package version 1.48.0 [33]. An adjustable number of top genes by variance are extracted and then clustered with the complete linkage method and plotted using the pheatmap package version 1.0.12 [34]. Results of the differential expression analysis are presented in a volcano plot generated with the EnhancedVolcano package version 1.4.0 [35]. Lists containing the results are exported as csv-files.

The pipeline is implemented as an R Shiny^®^ web application using the R packages shiny [36] and shinydashboard [37]. The Shiny^®^ web application is provided with the R package “tRomics” under the GPLv3 open-source license. It is publicly available via the URL https://github.com/miracum/clearly-tromics.

The application provides an interactive and easy-to-use graphical user interface (GUI) with options to define important settings manually, such as the definition of the design formula and sample comparison of interest. Moreover, the generated plots and result lists can be downloaded from the GUI in a publication-ready quality.

As the raw read count analysis is a standard process and can be performed with the web application, there are several possibilities for downstream analysis of DEGs depending on the researcher questions and experimental settings which can be done within and outside of R. We focus on functional enrichment and molecular interactome analysis with respect to miRNA regulation.

For the functional enrichment analysis, DEGs (logFC > 1.5, <−1.5; adj. *p* < 0.05; downloaded as csv-Files from the GUI application) were analyzed using g:Profiler web tool [1] and relevant enriched terms plotted using ggplot2 package version 3.3.0 [31] in R version 3.6.3. Furthermore, the DEGs were extended to their interaction partners by mapping them to the whole rat interactome downloaded from the International Molecular Exchange (IMEx) database [38] (accessed May 2020; 4896 nodes and 13,819 interactions; Table S4). The resulting molecular interactome was screened for miRNA-interaction partners using the miRTarBase [39] implemented in the Cytoscape (version 3.8.0) plugin CyTargetLinker version 4.1.0 [40].

### 4.3. Luciferase Reporter Assay

Prediction of miRNA-21-5p binding sites at rat *RELA* 3′-UTR was conducted with miRTarBase [39] revealing three potential miRNA-21-5p binding sites. A fragment of *RELA* 3′-UTR was PCR-amplified from rat cDNA applying *Spe*I and *Hind*III restriction sites integrated to primer sequences. Prediction of miRNA-223-3p binding towards rat *VIM* 3′-UTR was conducted with TargetScan [41] revealing one potential miRNA-223-3p binding site. The respective fragment of *VIM* 3′-UTR was PCR-amplified from a rat cDNA applying *Spe*I and *Hind*III restriction sites integrated to primer sequences. After restriction digest the respective fragment was subcloned to the pmiR report plasmid (Promega) and sequenced for appearance of correct miRNA binding sites. The resulting reporter plasmid was then co-transfected with 30 nM control miRNA or miRNA-21-5p or miRNA-223-3p and a normalizing beta-Gal expressing plasmid (Promega) to HEK293 cells for 24 h before validation of luciferase and beta-galactosidase activity.

## Supplementary Materials

Supplementary materials can be found at https://www.mdpi.com/1422-0067/21/13/4727/s1. Figure S1. Overview of the RNA-Seq analysis. (**Top**) Overview of the PCA analysis. (Middle) Overview of the MDS analysis. (**Bottom**) The volcano plot shows the differential expression analysis of the comparison lyco-s vs. buf-s; Figure S2. Mapping of the DEGs to the rat interactome results in a molecular interactome of 423 nodes and 113 edges (each node represents a DEG, edges interactions; color scaling shows the deregulation of genes (blue: downregulated, red: upregulated)); Table S1. Differential expression analysis of the RNA-Seq dataset; Table S2. Functional enrichment analysis of the deregulated genes after treatment with lyco-s using g:Profiler; Table S3. Differential expression analysis of the miRNA-Seq dataset after treatment with lyco-s; Table S4. Whole rat interactome downloaded from the IMEx database.
