# Supplementary material for: Integrative Bioinformatic Analyses of Global Transcriptome Data Decipher Novel Molecular Insights into Cardiac Anti-Fibrotic Therapies
Source: Int J Mol Sci. 2020 Jul 2;21(13):4727. doi: 10.3390/ijms21134727 (PMC7370212; doi:10.3390/ijms21134727)
Supplement: Supplementary file 1 [file ijms-21-04727-s001.zip › ijms-819271-supplementary final/supplementary_figures.pdf]

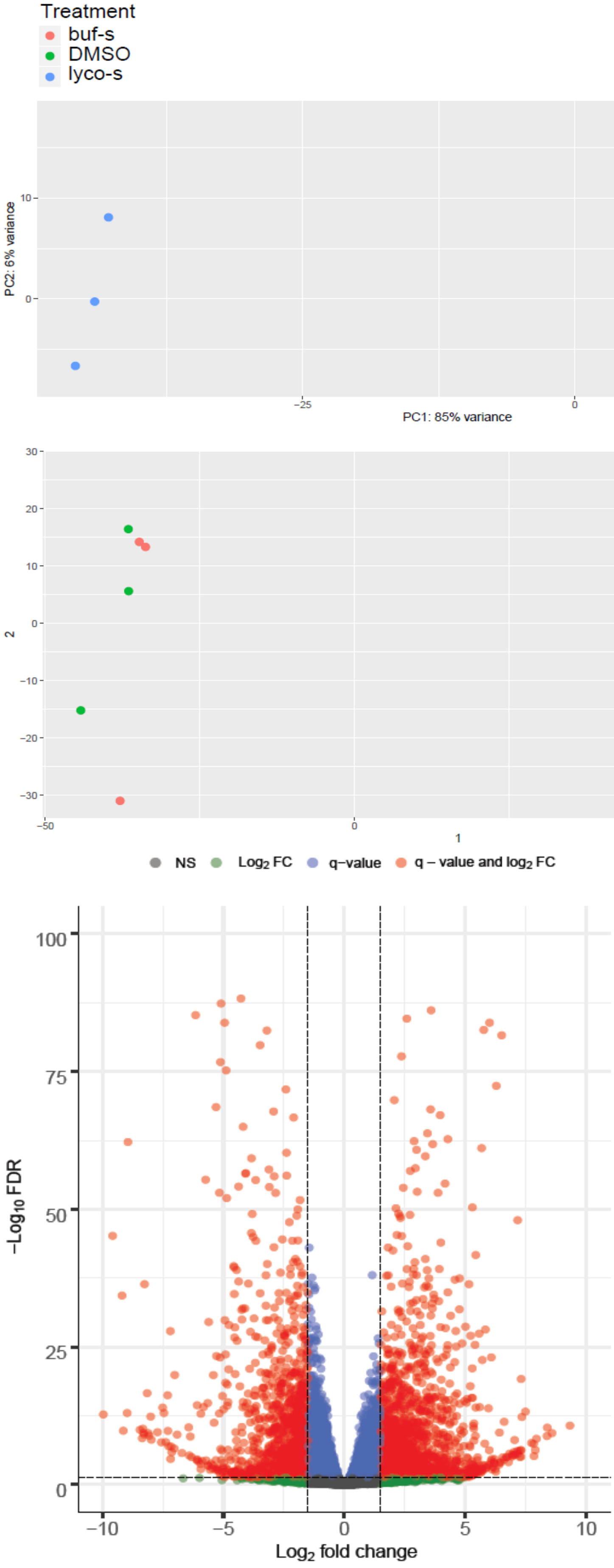


**Figure S1.** Overview of the RNA-Seq analysis. (Top) Overview of the PCA analysis. (Middle) Overview of the MDS analysis. (Bottom) The volcano plot shows the differential expression analysis of the comparison lyco-s vs. buf-s

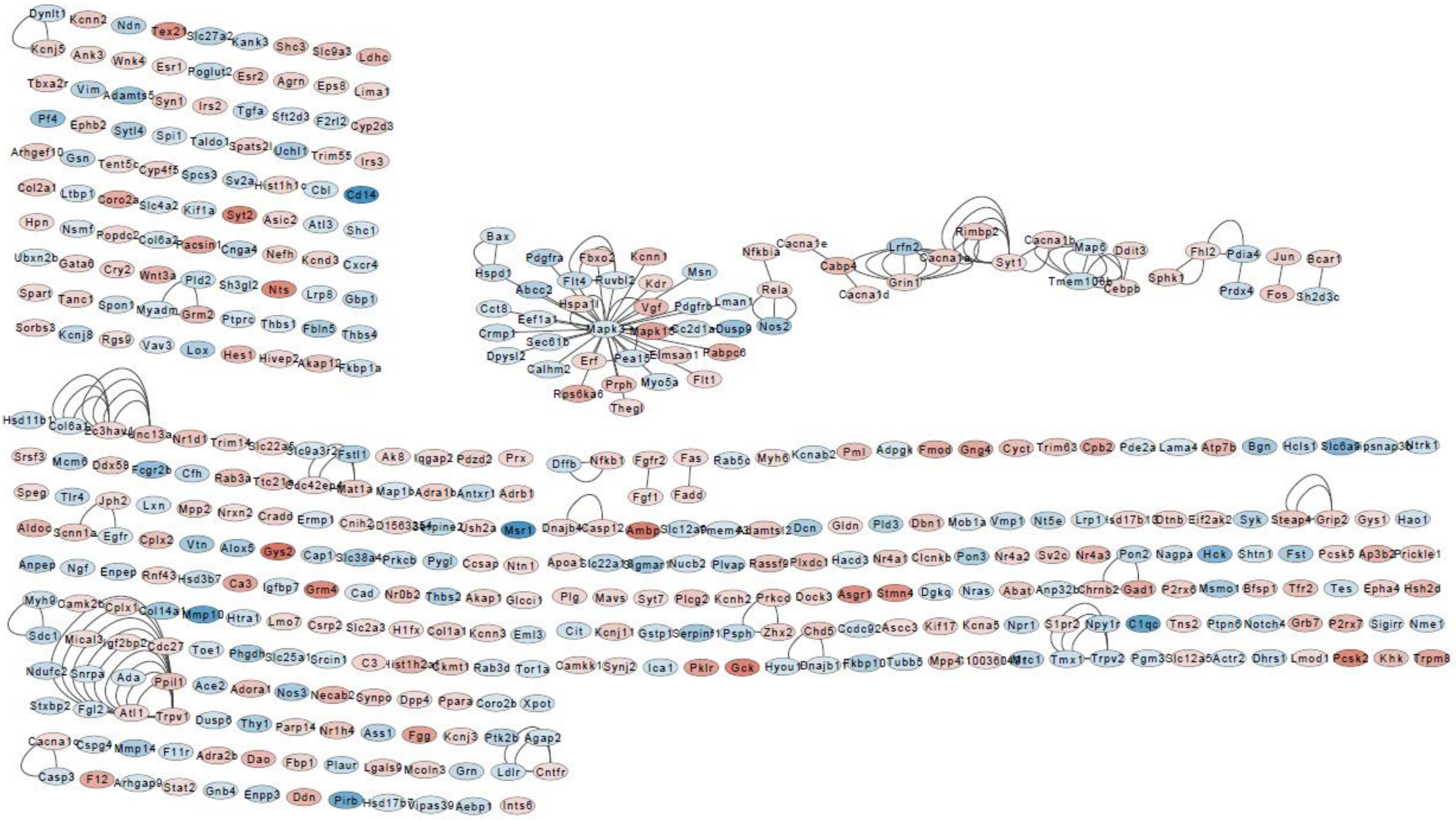

**Figure S2.** Mapping of the DEGs to the rat interactome results in a molecular interactome of 423 nodes and 113 edges (each node represents a DEG, edges interactions; color scaling shows the deregulation of genes (blue: downregulated, red: upregulated))
